# Necrosis Driven Triglyceride Synthesis Primes Macrophages for Inflammation During *Mycobacterium tuberculosis* Infection

**DOI:** 10.3389/fimmu.2018.01490

**Published:** 2018-07-03

**Authors:** Neetika Jaisinghani, Stanzin Dawa, Kaurab Singh, Ananya Nandy, Dilip Menon, Purva Deepak Bhandari, Garima Khare, Anil Tyagi, Sheetal Gandotra

**Affiliations:** ^1^Chemical and Systems Biology Group, CSIR-Institute of Genomics and Integrative Biology, New Delhi, India; ^2^Academy of Scientific and Innovative Research (AcSIR), New Delhi, India; ^3^Department of Biochemistry, University of Delhi South Campus, New Delhi, India; ^4^Guru Gobind Singh Indraprastha University, New Delhi, India

**Keywords:** necrosis, tuberculosis, macrophage, triglyceride, inflammation

## Abstract

Pulmonary tuberculosis (TB) exhibits granulomatous inflammation, a site of controlling bacterial dissemination at the cost of host tissue damage. Intrigued by the granuloma type-dependent expression of inflammatory markers in TB, we sought to investigate underlying metabolic changes that drive amplification of inflammation in TB. Here, we show an association of higher inflammation in necrotic granulomas with the presence of triglyceride (TG)-rich foamy macrophages. The conspicuous absence of these macrophages in solid granulomas identified a link between the ensuing pathology and the metabolic programming of foamy macrophages. Consistent with *in vivo* findings, *in vitro* infection of macrophages with *Mycobacterium tuberculosis* (Mtb) led to increase in TG synthesis only under conditions of ~60% necrosis. Genetic and pharmacologic intervention that reduced necrosis prevented this bystander response. We further demonstrate that necrosis independent of Mtb also elicits the same bystander response in human macrophages. We identified a role for the human enzyme involved in TG synthesis, diacylglycerol *O*-acyltransferase (DGAT1), in this phenomenon. The increased TG levels in necrosis-associated foamy macrophages promoted the pro-inflammatory state of macrophages to infection while silencing expression of diacylglycerol *O*-acyltransferase (DGAT1) suppressed expression of pro-inflammatory genes. Our data thus invoke a role for storage lipids in the heightened host inflammatory response during infection-associated necrosis. Our data provide a functional role to macrophage lipid droplets in host defense and open new avenues for developing host-directed therapies against TB.

## Introduction

Tuberculosis (TB) is the major cause of worldwide mortality due to any single infectious agent (1). Inflammation in TB plays a dual role—onset of the innate inflammatory response is crucial for the recruitment and activation of macrophages while dysregulation of this response leads to disease exacerbation (2). The inflammatory response in TB also contributes to tissue necrosis and cavitation which is the characteristic end-stage tissue damage (3, 4) leading to chronic impairment of pulmonary function (5, 6). Moreover, the heterogeneity of the inflammatory response in human TB granulomas within the same tissue suggests that local alterations in macrophage function might lead to gradation of responses (7, 8). Understanding the trajectory of macrophage fate during infection holds key to targeting immunopathogenesis of an active TB infection.

We sought to investigate if local metabolic alterations can alter the inflammatory response of human macrophages to the bacilli. The importance of lipid metabolism in infection induced inflammatory response has emerged from benefits of treatments that target lipid metabolism in preclinical models of TB (9). These models have revealed that nuclear receptors responsive to lipids, such as peroxisome proliferator-activated receptor-γ and liver X receptors (LXRα and LXRβ), protect against TB by regulating both pro- and anti-inflammatory pathways (9, 10). In addition, targeting the synthesis of lipid mediators of inflammation derived from arachidonic acid, by the non-steroidal anti-inflammatory drug Ibuprofen, has shown promise toward limiting pathology in the mouse model of infection (11). Given that triglyceride (TG) is a major lipid in human TB granulomas (12) and acts as a sink of fatty acids and signaling mediators such as diacylglycerides, phosphatidic acid, and phospholipids, its role in TB infection needs to be addressed. Understanding the mechanistic basis for differentiation of macrophages to TG-rich foamy macrophages during infection would be the first step in addressing whether TGs are important in control of inflammation and essential for bacterial survival.

In an attempt to understand the relationship of local inflammatory responses and TG accumulation in foamy macrophages, we established an association between high TNFα expression and appearance of TG-rich foamy macrophages in guinea pig granulomas. Both were conspicuously present in regions surrounding necrosis. Using a combination of biochemical and genetic tools we demonstrate that, during *Mycobacterium tuberculosis* (Mtb) infection of macrophages, necrosis is key to the increase in TG synthesis and accumulation in bystander cells. Importantly, we describe an *ex vivo* model of necrosis-associated foamy macrophages (NAFMs). These lipid-laden foamy macrophages mount a heightened inflammatory response to infection which can be suppressed by targeting diacylglycerol *O*-acyltransferase (DGAT1). Together, our data demonstrate that necrosis-associated inflammatory responses in TB may be mediated through bystander TG accumulation.

## Materials and Methods

### Cell Culture and Reagents

THP1 monocytes obtained from ECACC were cultured in RPMI 1640 (with glutamax, high glucose, HEPES, and sodium pyruvate) from Himedia with 10% FBS from Himedia. Phorbol 12-myristate 13-acetate (PMA), orlistat, T863 (2-((1,4-trans)-4-(4-(4-Amino-7,7-dimethyl-7H-pyrimido[4,5-b][1,4]oxazin-6-yl)- phenyl)cyclohexyl)acetic acid), chlorpromazine (CPZ) (C8138), and C75 (4-Methylene-2-octyl-5-oxotetrahydrofuran-3-carboxylic acid) were obtained from Sigma, IM54 (ALX-430-137-M001) was obtained from Enzo life sciences. THP1 monocyte culture media were analyzed for *Mycoplasma* contamination using a luminescence based kit (*Lonza*, LT07-118). THP1 cell line authentication was performed at Lifecode technologies Private Limited using 10 genetic loci *viz*: TH01, D21S11, D5S818, D13S317, D7S820, D16S539, CSF1PO, AMEL, vWA, and TPOX. The sample genotypes were queried against reference genotypes available in ATCC and DSMZ^®^ reference cell line STR databases to authenticate sample identity. Mtb H37Rv and *Mycobacterium bovis* BCG Tokyo were kind gifts from Dr. Vivek Rao, ΔRD1, and its wild-type control were kind gifts of Dr. David Sherman and Dr. Krishnamohan Atmakuri. Mycobacterial strains were transformed with the plasmids expressing fluorescent reporter proteins. pCherry3 was a kind gift from Dr. Tanya Parish (addgene#24659), pMV261-emGFP was a kind gift from Dr. Vivek Rao, and pTEC18 was kind gift from Dr. Lalita Ramakrishnan (addgene#30177).

### Guinea Pig Lung Histology Analysis

Guinea pigs were infected with Mtb H37Rv *via* the respiratory route in an aerosol chamber (Inhalation exposure system, Glas-Col, IN, USA) at ~30 cfu per animal. At week 4 and week 10 post infection, three guinea pigs per time point were euthanized by using CO_2_ asphyxiation. After dissecting the animals, three lobes (right caudal, middle, and cranial) of each lung were fixed in 10% buffered formalin. The left caudal portion of the lung was used for the enumeration of the bacillary load. For this, the left caudal portion was weighed and homogenized in 5 ml saline in a polytron PT2100 homogenizer followed by plating on 7H11(OADC) media. Colony counts were extrapolated for the whole lung based on weight of the left caudal lobe and the entire lung for each animal. Macroscopic granulomatous tissues were cut from the lung and kept in 20% sucrose solution overnight at 4°C followed by cryosectioning at section depth of 5 µm using *Leica Cryostat CM 1850*.

#### Hematoxylin and Eosin Staining

Sections on the slides were rehydrated using water for 5 min. The slides were then dipped in hematoxylin solution (1:10 Delafield’s Hematoxylin solution) for 5 min and then rinsed in water. The slides were then stain intensified by placing in ammonia solution (0.08%) for 1 min, followed by rinsing in water for 5 min. The sections were then equilibrated in 95% ethanol solution followed by dipping them in eosin stain (1%) for 15 s. The sections were then dehydrated in 95% and 100% ethanol for 2 min in each solution, and finally rinsed in water for 5 min. The slides were then cleaned and mounted using 20% glycerol.

#### Hematoxylin and Oil Red O Staining

The sections were first dipped in a prewarmed Oil Red O solution (0.18% in 60% isopropanol) for 1 h at 60°C, then rinsed twice with water for 5 min each. The slides were then dipped in hematoxylin solution for 5 min, rinsed, and mounted in 20% glycerol. Images were acquired using Leedz Microimaging 5 MP camera attached to a Nikon Ti-U microscope. Analysis was performed by four individuals independently including a trained pathologist.

### Immunofluorescence of Guinea Pig Lung Sections

Serial sections to those used for oil red O staining were used for TNFα and MAC1 immunostaining. TNFα immunostaining: sections were blocked with 5% bovine serum albumin (BSA) for 1 h, followed by overnight incubation with anti-TNFα antibodies (1:50, ab1793). Alexa-633 tagged secondary antibody was used for detecting the primary antibody reactivity, followed by DAPI counterstaining. Images were acquired using a laser scanning microscope in the widefield mode. MAC1 immunostaining: cryosections were incubated with 0.25% Tween-20 in 1× PBS for 10 min for permeabilization, sections were rinsed thrice with 1× PBS for 10 min each. Sections were incubated with primary antibody, 1:50 dilutions, Anti-MAC [MAC387] (ab22506) for 2 h at room temperature after blocking with 1% BSA for 1 h. Sections were then incubated with Alexa 633 conjugated secondary antibody (1:200 dilutions, Life Technologies, A21052) for 30 min at room temperature, sections were washed well with 1× PBS thrice for 10 min each. Sections were counterstained with BODIPY 493/505 for 1 h at room temperature. Coverslips were mounted on the sections using Prolong Diamond Antifade mountant with DAPI. Images were acquired using Leica SP8 Confocal microscope.

### Extraction of Lipids From Granulomatous Lesions of Guinea Pig Lungs

Visible lesions from infected lungs and anatomically comparable regions from uninfected lungs were dissected out at indicated time points and then weighed. These tissues were then sonicated in 600 µl of chloroform:methanol (2:1) at 60°C in a water bath sonicator. Lipids were extracted using the modified Bligh and Dyer method by first making the sonicated extract to 1,200 µl of chloroform:methanol (1:2) by adding 600 µl of methanol. One-fourth volume of 50 mM citric acid, half volume of water, and one-fourth volume of chloroform were added and vortexed. This was then centrifuged at 9,600 *g* for 10 min and then the lower phase taken and dried. The dried lipid extracts were weighed and then resuspended in chloroform:methanol (2:1) such that the concentration of the extract was 0.1 mg/µl and extracts corresponding to 0.5 mg were loaded on TLC. TLCs were developed in 4°C in the solvent system hexane:diethyl ether:acetic acid (70:30:1) for neutral lipids.

### *DGAT1* Knockdown

Lentiviral particles for *DGAT1* knockdown were obtained from Transomic technologies (shRNA sequences used DGAT1 pZIP-hEF1 alpha zsgreen: TGCTGTTGACAGTGAGCGCCCTACCGGGATGTCAACCTGATAGTGAAGCCACAGATG TATCAGGTTGACATCCCGGTAGGATGCCTACTGCCTC GGA).

50,000 THP1 monocytes were transduced with lentiviral particles at multiplicity of infection (MOI) 10 for 24 h. Transduced cell lines were maintained for stable cell line generation in puromycin at 0.6 µg/ml for about 3–4 weeks. Knockdown efficiency was checked using qRT-PCR and also validated using TG analysis from TLC.

### RNA Isolation and qRT-PCR Analysis

1.2 million cells were used for isolation of RNA from a single replicate of a condition, with three replicate wells per experiment. Cells were infected at MOI 50 for 3 h. The monolayers were washed three times with media and replaced with RPMI media supplemented with 10% FBS. At 24 h post infection, cells were scraped in TRIzol (Ambion) or RNAzolRT (Sigma). RNA was extracted into the aqueous phase, precipitated, and further purified using the Qiagen RNeasy kit or by organic precipitation. The purified RNA was DNase treated using Turbo DNAse (*Ambion, AM2238*) for 1 h at 37°C. 1 µg of purified RNA was used for cDNA synthesis (*Invitrogen, 18080-093*). cDNA was diluted 10 times and 2 µl of the diluted cDNA was used for expression analysis of selected genes by qRT-PCR (*Roche, LightCycler Sybr green master mix, 04707516001*). Primer sequences used for qRT-PCR were as follows:

**Table:** 

Gene name	Primer	Sequence
*PPIA*	Forward	ATGCTGGACCCAACACAAAT
	Reverse	TCTTTCACTTTGCCAAACACC
*DGAT1*	Forward	GCCTGCAGGATTCTTTCTTC
	Reverse	AGACATTGGCCGCAATAACC
*TNFA*	Forward	CCCCAGGGACCTCTCTCTAATC
	Reverse	GGTTTGCTACAACATGGGCTACA
*IL1B*	Forward	CTCGCCAGTGAAATGATGGCT
	Reverse	GTCGGAGATTCGTAGCTGGAT

### Lipid Extraction From Macrophages (Modified Bligh and Dyer Method)

For making a total lipid extract from THP1 monocyte-derived macrophage and human MDMs, the cells were first washed with PBS twice and then lysed in 1% Triton X100. After lysis, four volumes of methanol:chloroform (2:1) was added, and the lysate was vortexed. One volume of 50 mM citric acid, one volume of water, and one volume of chloroform were added and vortexed. This was then centrifuged at 10,000 *g* for 10 min, and then the lower phase isolated and dried. The dried lipid extract was then resuspended in chloroform:methanol (2:1) and loaded on TLC. TLCs were developed in 4°C in the solvent system hexanes:diethyl ether:acetic acid (70:30:1) for neutral lipids. For cholesterol estimation, the TLCs were pre-run in chloroform:methanol (90:10) followed by baking them at 110°C for 30 min. After spotting the samples and standards, the TLCs were developed sequentially in solvent system 1: chloroform:methanol:water (65:25:4) and solvent system 2: hexane:diethyl ether:acetic acid (70:30:2).

For visualization of unlabeled lipids on TLC, the TLCs were stained either using 10% copper sulfate (w/v) in 8% phosphoric acid (v/v) solution or phosphomolybdic acid (10% in ethanol) solution followed by charring at 150°C. Quantification of unlabeled lipid spots was done using ImageJ.

### Measurement of Cellular TG Synthesis

THP-1 monocytes were differentiated into macrophages using 100 µM PMA at a density of 0.6 million cells/ml for 24 h, followed by 2 days in media without PMA. Subsequently, cells were infected with Mtb H37Rv at respective MOI for 24 h, in the presence of general lipase inhibitor orlistat (13) and 1 μCi/ml C^14^ oleic acid (*ARC0297*) or 1 µM BODIPY558/568C12 as described previously (14). This duration of treatment and concentration of orlistat was not found to affect mycobacterial viability. Total cellular lipids were extracted at 24 h post addition of pulse using the modified Bligh and Dyer method, dried, and then analyzed using thin layer chromatography. TLCs were scanned using the Typhoon Scanner and densitometry analysis performed using Image Quant 5.2. Fatty acid incorporation into TG during necrotic cell supplement (NcS) stimulation was measured in the same manner as explained above.

### Cell Death Assays

#### Annexin V and Propidium Iodide (PI) Staining

THP1 macrophages seeded at a density of 0.6 million cells/ml were infected with Mtb, MtbΔRD1, or *M. bovis* BCG at indicated MOI. Cell death by apoptosis or necrosis in macrophages infected with different strains was quantified by Alexa Flour^®^ 488 Annexin V/Dead cell Apoptosis kit from Invitrogen. This was done at 8 h post infection rather than 24 h as in all other experiments so as to reduce likelihood of secondary necrosis being measured along with primary necrosis. This also allowed sufficient number of cells to be left for accurate quantification of % annexin and PI positivity in case of wild-type H37Rv without analysis being skewed by the decrease in total number of cells. Monoloyers were washed with 1× Annexin binding buffer twice, and then stained with 10 µl Alexa Flour 488 Annexin V and 1 µl of 10 mg/ml PI in 100 µl of 1× Annexin binding buffer for 15 min at room temperature. Monolayers were again washed with buffer twice and fixed with 4% formaldehyde for 30 min at room temperature. The coverslips were washed with PBS and mounted using ProLong Antifade with DAPI. Wide field z-stacks were acquired using Leica TCS SP8 (40× objective) followed by counting annexin V or PI positive cells manually from five images for each group.

#### Lactate Dehydrogenase Release Assay

Culture supernatants from uninfected and infected THP1 macrophages were used for LDH activity assays using Cytotoxicity detection kit from Roche. Cell death was enumerated as a percentage of cells dying in treatment groups as compared to cell death in cells treated with Triton X100 (lysis buffer in kit).

#### Cell Death Estimation by Cell Counts

THP1 macrophage monolayer was fixed with 4% formaldehyde for 15 min at room temperature followed by washing with PBS three times for 5 min each. Cells were then stained with 2% DAPI solution for 30 min at room temperature followed by washing with PBS three times for 5 min each. 10 images for each well were acquired in EVOS Floid cell imaging system using the UV lamp. Images from different groups were subjected to cell counts using Volocity software from Perkin Elmer.

### Supernatant Transfer Experiments

#### Donor Cell Preparation

THP1 macrophages were seeded in 12-well plates at a density of 0.6 million cells per well, and their lipids were labeled using BODIPY 558/568 C12 overnight. Post 16 h of labeling, they were washed with PBS to remove the extracellular label and then infected at MOI 50 for 24 h in the presence or absence of IM54 (20 µM). After 24 h, the supernatant was collected and transferred to recipient cells thought a 0.45 μm transwell filter (Milipore). *Recipient cells*: THP1 macrophages were seeded on coverslips in 12-well plates. Third day after differentiation with as above, medium was changed to supernatant from infected macrophages (for each well supernatant for three wells were combined) through the filter. CPZ (10 µM) was added to recipient cells during treatment. After 48 h of treatment, coverslips were fixed and stained with BODIPY493/503, followed by confocal microscopy.

### Immunofluorescence

Cells were fixed using neutral buffered, methanol-free 4% formaldehyde, permeabilized using 0.5% Saponin, blocked with 3% BSA, and incubated overnight with primary antibodies. Primary antibodies used for immunostaining: CD44 (BD Pharmingen 550392) and Adipophilin (ADRP) (Progen 610102). Secondary antibodies used for detection were highly cross adsorbed antibodies conjugated to Alexa fluor dyes (Invitrogen).

### Lipid Droplet (LD) Imaging and Analysis

Lipid droplet size distribution in THP1 cells expressing either non-targeting shRNA or shRNA against *DGAT1* was performed using InCell6000 (GE) in the confocal mode. Two days post PMA removal, they were treated with 1 µM BODIPY665/676 or LipidTox Deep Red to visualize LDs. 10 min post staining, cells were imaged. LDs were identified using built-in vesicle identification tools with additional segmentation restrictions to allow object separation. Analysis was done by compartmentalization of LDs per cell using built-in scripts. For all other experiments, THP-1 monocytes were seeded with 100 µM PMA on glass coverslips of 0.17 mm thickness for 24 h, followed by 2 days in media without PMA. In all experiments, macrophages were fixed using 4% methanol-free formaldehyde for 30 min. Fixed coverslips were washed with PBS and then stained using BODIPY493/503 (Invitrogen, D3922) solution at 10 µM for 1 h at room temperature, followed by three washes in 1× PBS. After staining, the coverslips were mounted on slides and sealed. Confocal z-stacks of 0.30 mm thickness were taken using Leica TCS SP8. LD volume measurements were done based on BODIPY493/503 fluorescence using VOLOCITY (Perkin Elmer) image analysis software. Statistical significance was calculated from non-parametric Kruskal–Wallis test with a *post hoc* Dunn’s test for comparison of groups. Mean LD volume from multiple experiments was tested for significance using Student’s *t*-test. Mean cellular fluorescence (for either BODIPY493/503 or BODIPY558/568) was calculated using the Leica LAX 3.1.1 software; cell boundaries were made using a free hand selection tool and total fluorescence in the selected region of interest reported. Total fluorescence/cell from each experiment was taken and data from three to four such experiments were pooled.

### Measurement of Mean Bacterial Fluorescence Per Cell in Infected Macrophages

THP1 macrophages seeded at a density of 0.3 million cells/ml were infected with Mtb strains expressing mCherry-H37Rv and H37RvΔRD1, at indicated MOI for 24 h. After fixation using 4% methanol-free formaldehyde, macrophages were stained with BODIPY493/503 at 10 µM, Cell Mask (Invitrogen, C10046) at 1 µg/ml and DAPI at 1 µg/ml concentration for half an hour at room temperature. The wells were then washed with PBS three times for 5 min each and proceeded with imaging. Nine confocal z-stacks were acquired randomly for each well at 63× objective in IN CELL 6000 High content imager (GE Health care). Images were analyzed for estimation of mean fluorescence intensity of bacteria per cell was analyzed using IN CELL Developer toolbox. Cell boundaries and nuclei were marked using Cell mask and DAPI fluorescence. Mean fluorescence density of bacteria overlapping with the fluorescence in the Cell mask channel was quantified. This arbitrary fluorescence measure was background corrected and thereafter plotted as a surrogate measure for bacterial uptake per cell.

### THPM Necrosis and Media Preparation

Cells were scraped in media and then frozen at −20°C temperature followed by thawing at room temperature. This was repeated five times to make the NcS. NcS was then added on THP1 monocyte-derived macrophages at a density of 1.2 million necrosed cells/ml media for 8 days, changing media every 2 days with PMA addition at day 1 and day 5 post additions of NcS. The “normal media” control group was cultured in the same manner except necrotic cells were not added as supplement.

### Biochemical Estimations

Quantification of cholesterol, TG, and protein concentration in necrotic supplement and normal media was performed by specific colorimetric reactions for each of the analytes using COBAS INTEGRA 400 plus (Roche Diagnostics). Glucose estimation was performed using the anthrone method.

### Glucose Uptake Assays

14C-2-deoxyglucose (ARC 0112A) (1 μCi/ml) was added to differentiated THP1 macrophages in media or NcS for 5, 24, and 48 h. At these time points, cells were washed four times with media, and cell lysates were prepared. Cell lysates at each time point were added to 100 µl of scintillation cocktail (*Microscint PS, Perkin Elmer, 6013631*) in a white 96-well flat bottom plate and then read in the Perkin Elmer TopNXT Scintillation counter. Glucose uptake was plotted as ratio of radioactivity present in the cell lysate to the total radioactivity added.

### Cytokine Analysis

THP1 macrophages were cultured as described above for 8 days, followed by infection with Mtb at MOI 50 for 3 h at which point extracellular bacilli were removed. The monolayers were washed three times with media and replaced with RPMI media supplemented with 10% FBS. Cell culture supernatants were harvested at 24 h post infection and assayed for TNFα using an ELISA kit (eBioscience) or Milliplex human cytokine/chemokine bead panel (HCYTOMAG-60K).

### Isolation and Culture of Human PBMCs

Blood (9 ml) was drawn from five individuals with their consent in K3EDTA containing vacutainers. Blood was first diluted in RPMI to 30 ml which was then layered on top of 10 ml Histopaque (Sigma, 10771) and centrifuged at 500 *g* for 30 min at 20°C with acceleration 9 and deceleration 1. After centrifugation, the buffy coat was collected in a separate tube and washed with PBS once. To remove platelets from the pellet, the pellet was resuspended in RPMI and then layered on top of 5 ml FBS followed by centrifugation. For removal of RBCs, the pellet was resuspended in 9 ml of water for 10 s followed by addition of 1 ml of 10× PBS. Cells were collected by centrifugation and then allowed to differentiate into macrophages in RPMI media containing FBS or human pooled serum, with or without human M-CSF or GMCSF for 6 days. In between, at day 4, the monolayer was washed to remove non-adherent cells with PBS. Infections were done at day 8 post isolation. For TNFα release measurements, PBMCs were differentiated in 50 ng/ml GMCSF for 6 days, followed by 8 days in media or NcS in the presence of 10 ng/ml GMCSF.

### Intracellular Mycobacterial Growth Assay

Macrophages were seeded in 48-well plates at a density of 0.6 million/ml and infected at MOI of 0.1 for 4 h, followed by removal of extracellular bacilli by 3 washed with PBS and addition of complete growth media. Cells were lysed in 100 μl of 1% Triton-X100 and serial dilutions plated on 7H10 OADC plates. Media was changed after every 48 h.

### Statistics

All statistics were computed using Prism. ANOVA and *t*-test were used for all parametric data wherein data from three to four independent experiments were combined. For testing statistical significance of change in median of the LD size (Figure 2C) and per cell signal of BODIPY558/568 (Figure 4C), Kruskal–Wallis test was used.

### Ethics Statement

The animals were housed and handled at the Department of Biochemistry, University of Delhi South Campus according to directives and guidelines of the Committee for the Purpose of Control and Supervision of Experiments on Animals (CPCSEA). Use of Mtb infected guinea pig lung tissue for this work was approved by the CPCSEA as per ethics proposal #IGIB/IAEC/14/15. Blood was drawn from healthy human volunteers with informed written consent as per approval #11dtd.March30th2015 of the Institutional Human Ethics Committee.

## Results

### Necrotic TB Granulomas Exhibit Heightened Inflammation Associated With Increased TG Accumulation

**Figure 1:**
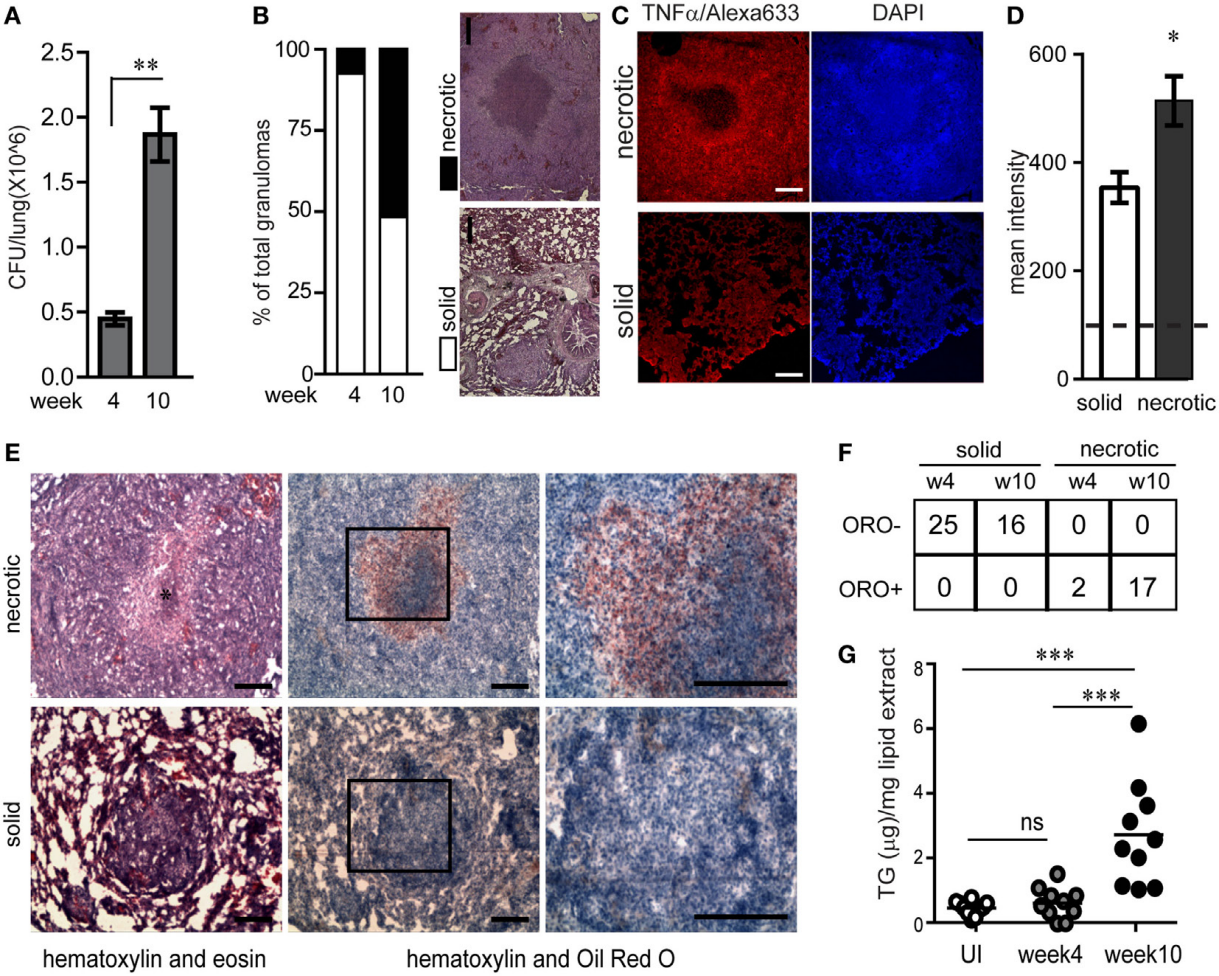
Necrotic tuberculosis granulomas exhibit increased TNFα production and spatial polarization of triglyceride-rich foamy macrophages. **(A)** CFU/lung per guinea pig (*N* = 3 per time point) at week 4 and 10 post aerosol infection with ~30 cfu/lung. **(B)** Percentage of solid and necrotic granulomas of total granulomas in tissue sections analyzed from three animals at each time point. Images are examples of a typical solid and necrotic granuloma stained with haematoxylin + eosin. Scale bar = 100 µm. **(C)** TNFα immunofluorescence in solid and necrotic guinea pig granulomas. Right panel shows nuclear staining with DAPI (scale bar = 250 μm). **(D)** Quantification of TNFα immunofluorescence as mean intensity/granuloma from four solid and three necrotic granulomas. Dashed horizontal line is the mean value from controls without primary antibody. **(E)** Sequential cryosections stained with haematoxylin + eosin (left panels) and haematoxylin + Oil red O (center and right panels) from a necrotic and solid granuloma. * indicates necrotic center filled with pyknotic nuclei. Scale bar = 100 μm. **(F)** Table showing number of oil red O negative (ORO^−^) and positive (ORO^+^) solid and necrotic granulomas at week 4 and week 10 post infection. **(G)** TG quantification from 10 individual macroscopic granulomas from a single animal at each time point, calculated using densitometry of thin layer chromatographs. Each circle represents TG from a single macroscopic granuloma or anatomically similar section from control animals. Line indicates mean TG of each group (*p < 0.05, **p < 0.01, ***p ≤ 0.001, ns, not significant). Also see Figure S1 in Supplementary Material.

The guinea pig model of pulmonary TB presents with the array of granulomas observed in human TB, including solid and necrotic granulomas which may be caseous, fibrocaseous, or cavitary lesions (12, 15, 16). To study how lesion types differ in their inflammatory nature and abundance of lipid-loaded foamy macrophages, we investigated lesions from infected guinea pigs at week 4 and 10 post aerosol infection with virulent Mtb strain H37Rv with ~30 bacilli/lung. By 4 weeks post infection, the bacterial burden per lung was found to be 4.1 × 10^5^ ± 1.7 × 10^5^ (Figure 1A). Bacterial burden increased further 4-folds by 10 weeks post infection (Figure 1A). At 4 weeks post infection 92.8% of the granulomas were non-necrotic (referred to as solid) while at week 10 approximately half of the granulomas were found to be necrotic, with amorphous eosin staining (Figure 1B) interspersed with pyknotic and karyorrhectic nuclei at the core (Figure S1A in Supplementary Material). These findings were consistent with bacterial burden associated necrosis in TB (17). Consistent with previous reports in humans (18), we observed distinctive staining for TNFα at the cellular cuff of the necrotic core (Figure 1C). By contrast, TNFα immunostaining in solid granulomas seemed to be more diffuse and in general lower compared to necrotic granulomas (Figures 1C,D). This suggested that perhaps exposure to necrosis would lead to the higher expression of TNFα.

Further histological analysis using oil red O staining revealed that necrotic granulomas also exhibited a cellular cuff of oil red O positive cells (Figure 1E). While 100% of necrotic granulomas were oil red O positive, this staining was conspicuously absent from solid granulomas (Figures 1E,F). These cells with higher abundance of neutral lipid content were confirmed to be macrophages using immunostaining for mac-1, a macrophage marker, and staining for BODIPY493/503, a neutral lipid fluorescent dye (Figures S1B,C in Supplementary Material). Lipid analysis from macroscopic granulomas verified the neutral lipid to be mainly TG (Figure S1D in Supplementary Material). TG content from macroscopic granulomas likely to be necrotic (week 10) was higher as compared to anatomically similar regions of lungs from animals that were infected for 4 weeks or were uninfected (Figure 1G). These data demonstrated that as granulomas evolved to develop necrosis, they exhibited presence of TG rich foamy macrophages proximal to the necrotic core. In addition, the presence of TG rich foamy macrophages correlated with higher local TNFα in the cellular cuff of the necrotic core.

### TG Levels and LD Abundance in THP1 Macrophages Is Dependent on *DGAT1* Expression

**Figure 2:**
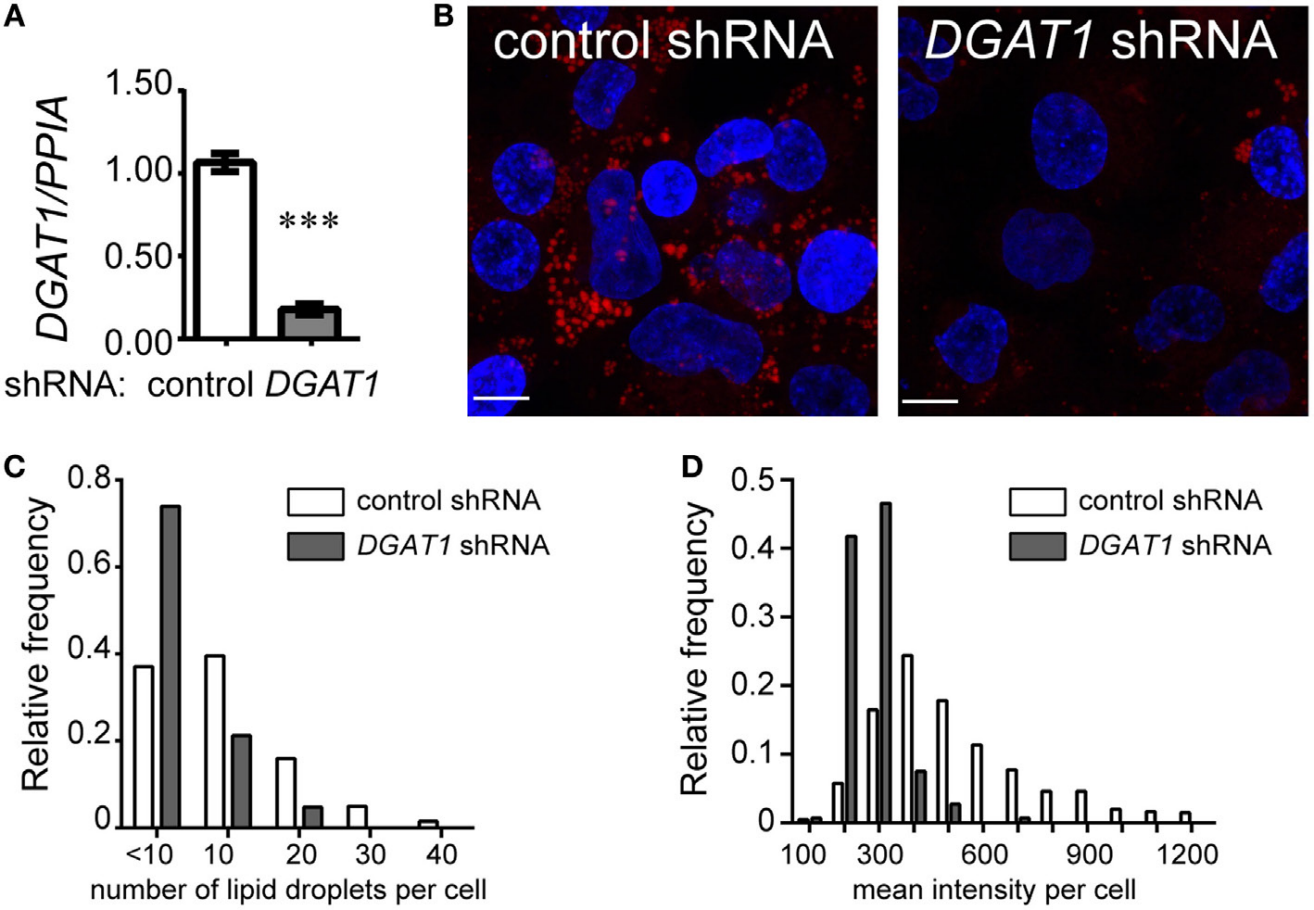
Triglycerides are the major neutral lipids in human THP1 macrophages. **(A)** *DGAT1* expression in stable THP1 cell lines derived upon transduction with lentiviruses expressing non-targeting (control) and *DGAT1-*targeting shRNA. Data are mean ± SEM from three independent experiments. **(B)** LipidTox (red) and DAPI (blue) stained control (non-targeting) and *DGAT1* shRNA expressing stable THP1 macrophages. Graphs representing distribution of lipid droplet size **(C)** and content (mean fluorescence intensity per cell) (D) from LipidTox stained images. Data are representative of 2 independent experiments. See also Figure S1 in Supplementary Material.

To further understand the possible relationship between TG metabolism and inflammation in TB infection, we sought to investigate whether TGs constitute the major neutral lipid in human macrophages. We used THP1 monocyte-derived macrophages for this purpose. TG storage can occur from either the MGAT pathway or the Kennedy pathway. Both pathways converge to generate diacylglycerol, which is then converted to TG by a diacylglycerol *O*-acyl transferase (19). Using lentivirus transduced THP1 cells expressing shRNA against the enzyme DGAT1, we achieved an 80–90% knockdown in *DGAT1* expression (Figure 2A) and 60% decrease in TG levels (Figure S1E in Supplementary Material). TG and other neutral lipids are bound by a monolayer of phospholipid membrane within cytosolic LDs (20). Neutral lipid staining using LipidTox also showed a decrease in abundance and size of LDs upon DGAT1 knockdown (Figures 2B–D). This demonstrated that in THP1 cells, majority of the neutral lipid is TG and its levels can be regulated efficiently using a *DGAT1* knockdown.

### Mtb Infection Increases Macrophage TG Synthesis During Necrosis

**Figure 3:**
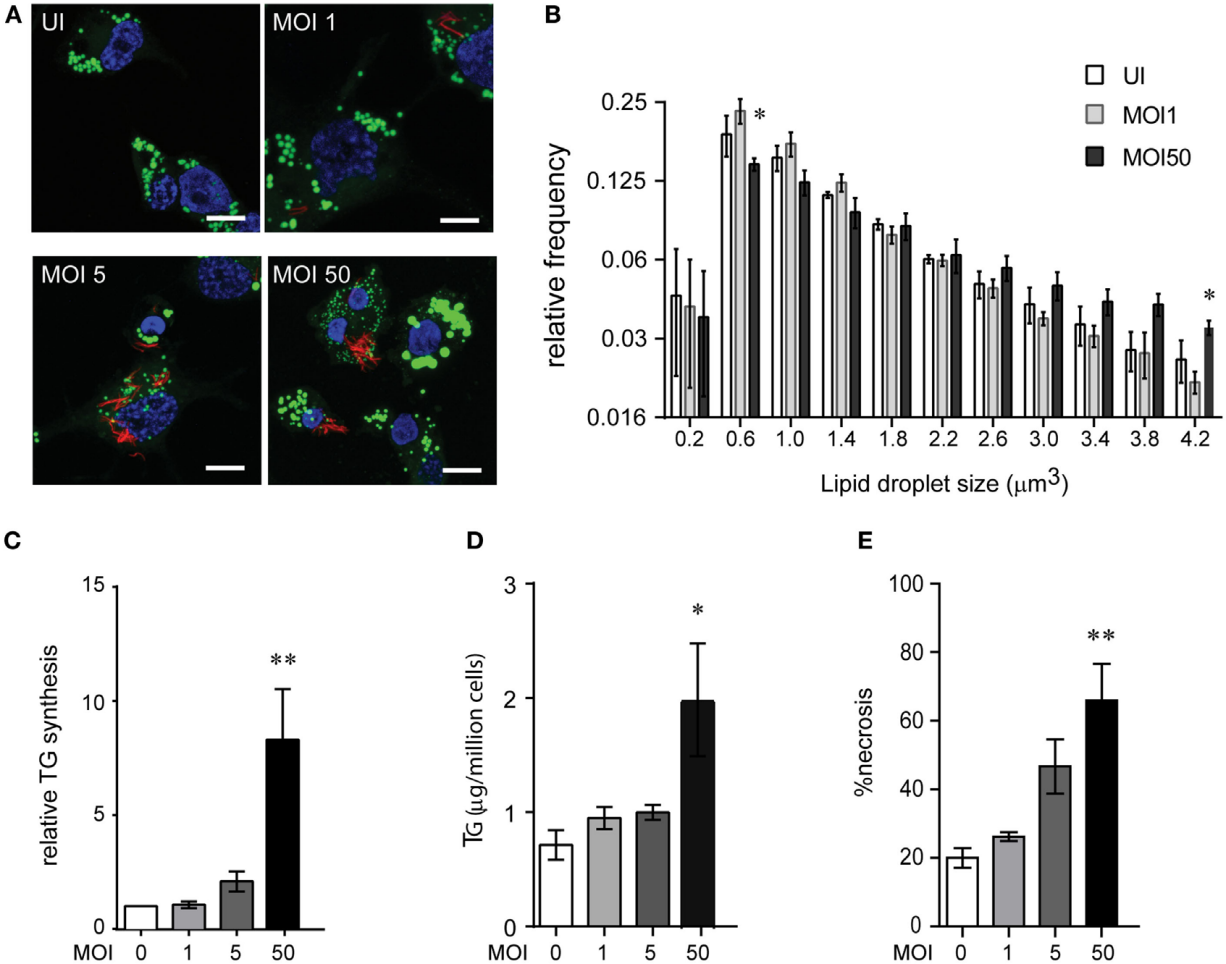
High multiplicity of infection (MOI) increases TG accumulation in human macrophages concomitant to necrosis. **(A)** BODIPY 493/503 (green) and DAPI (blue) stained THP1 macrophages at 24 h post infection with MOI 1. mCherry^+^ *Mycobacterium tuberculosis* can be seen in red. Scale bar = 10 μm. **(B)** Assessment of mean LD size estimated from five confocal z-stacks of each condition per experiment. Data are mean ± SEM from two to three independent experiments. **(C)** TG synthesis relative to uninfected cells, estimated in macrophages infected with indicated MOI at 24 h post infection and normalized to number of cells at the end of 24 h infection. Data are mean ± SEM from three independent experiments. **(D)** TG estimation in adherent cells at 24 h post infection with indicated MOI. Data are mean ± SEM from three independent experiments. **(E)** Quantification of cell death measured by LDH activity and represented as % of necrosis relative to detergent lysed cells. Data are mean ± SEM from three experiments. One-way ANOVA followed by Dunnett’s test derived *p*-values are indicated only for the significantly different groups relative to uninfected cells (**p < 0.01, and *p < 0.05). Also see Figure S2 in Supplementary Material.

We investigated if Mtb infection itself increases TG synthesis during macrophage infection. To understand how acute Mtb infection alters host total lipid content, we measured the size of LDs in PMA differentiated THP1 macrophages upon Mtb infection using BODIPY493/503. Mtb infection of THP1 macrophages for 24 h at a MOI of one (MOI 1^24h^) or five (MOI 5^24h^) led to infection of approximately 80% cells with at least one bacterium per cell. However, neither of these conditions led to an increase in size or content of LDs (Figures 3A,B). Human peripheral blood monocyte-derived macrophages, similar to THP1 macrophages, also did not show an increase in LD size or lipid content under the same infection conditions (Figures S2A–C in Supplementary Material). However, an increase in MOI to 50 led to a shift in the proportion of LDs of larger size (Figures 3A,B). Consistent with an increase in LD size, we observed an increase in TG synthesis (Figure 3C) and absolute levels of TG (Figure 3D) under MOI 50^24h^ condition of infection while no increase was observed at MOI 1^24h^ and MOI 5^24h^.

Incident to the increase in TG synthesis at MOI 50^24^ was an increase in cell death from 40% to over 60% (Figure 3E; Figure S2D in Supplementary Material). Therefore, the increase in TG levels could be a bystander response of remaining viable cells to necrosis. TG content in the remaining adherent cells revealed a linear correlation with the relative necrosis in that well (*R*^2^ = 0.59, *p* = 0.0002) (Figure S2E in Supplementary Material). Consistent with our data on guinea pig TB granulomas, increased TG synthesis and accumulation in THP1 macrophages in infection conditions with increased cell death suggested necrosis as a key player in foamy macrophage formation during Mtb infection.

### Genetic and Pharmacological Inhibition of Necrosis Prevents TG Synthesis in Bystander Macrophages During Infection

**Figure 4:**
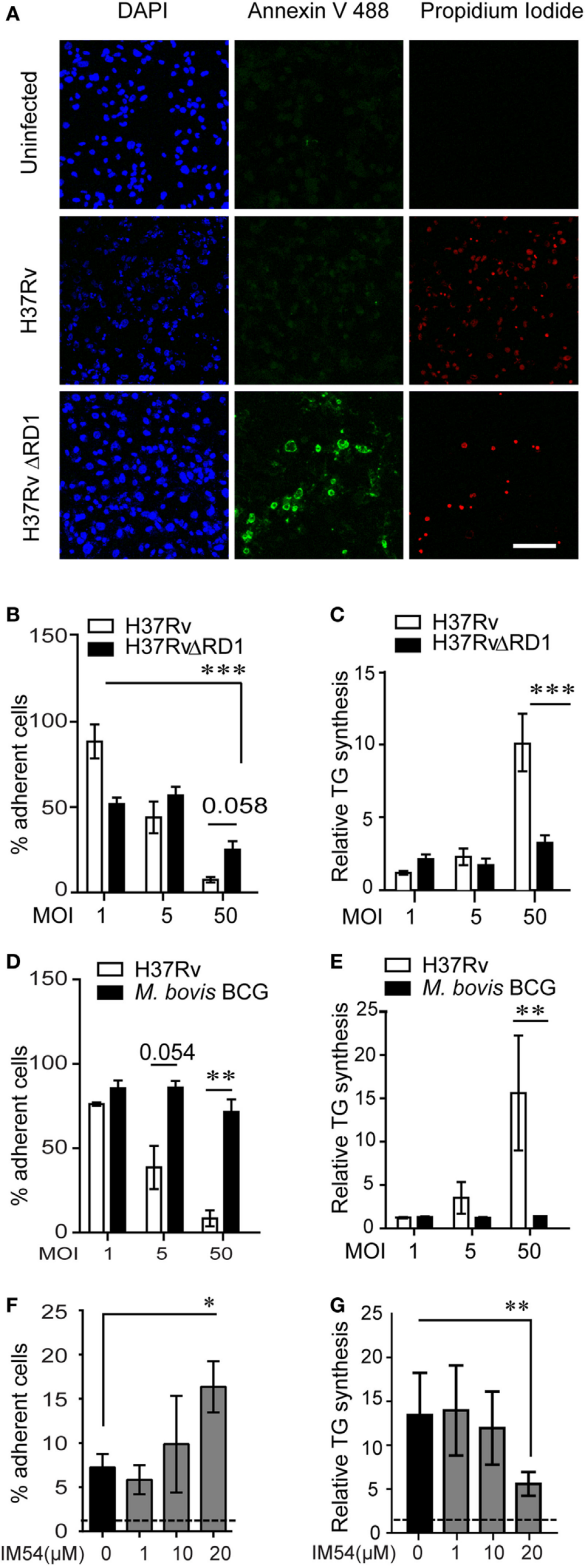
Inhibition of necrosis during *Mycobacterium tuberculosis* infection prevents TG accumulation in bystander macrophages. **(A)** Representative images of Annexin V and propidium iodide staining of uninfected and infected THP1 macrophages at 8 h post infection with the indicated strains at multiplicity of infection (MOI) 50. Scale bar = 100 μm. Graphs in **(B,D)** represent percentage of THP1 macrophages remaining adherent at 24 h post infection with H37Rv, H37RvΔRD1 **(B)**, or *Mycobacterium bovis* BCG **(D)** at indicated MOI as a percentage of uninfected control. Data are mean ± SEM from three independent experiments. Quantification of TG synthesis relative to uninfected cells at 24 h post infection with H37Rv, H37RvΔRD1 **(C)**, or M. *bovis* BCG **(E)** at indicated MOI. Data are mean ± SEM from three independent experiments. **(F)** Graph represents percentage of THP1 macrophages remaining adherent at 24 h post infection with H37Rv at MOI 50 in the presence of increasing concentration of IM54 as a percentage of uninfected control. Data are mean ± SEM from three independent experiments. **(G)** Quantification of relative TG synthesis in THP1 macrophages at 24 h post infection with H37Rv at MOI 50 in the presence of increasing dosage of IM54. Data are mean ± SEM from three independent experiments. *t*-test derived *p*-values are indicated (***p < 0.001, **p < 0.01, and *p < 0.05). Also see Figure S3 in Supplementary Material.

The mycobacterial pathogenicity associated locus, *region of difference 1* (RD1), has been shown to be involved in inducing atypical cellular necrosis (21–23) *via* a bacterial contact-dependent membrane deformation (24). The vaccine strain *M. bovis* BCG harbors a deletion of the RD1 locus (25) and is known to induce apoptosis rather than necrosis in macrophages (26, 27). Consistent with literature, we also observed Mtb infection to cause necrosis instead of apoptosis of THP1 macrophages with poor Annexin V staining (1–15%, *N* = 3) and efficient PI staining (20–85%, *N* = 3) within 8 h of infection at MOI 50 (Figure 4A; Figures S3A,B in Supplementary Material). In comparison, infection with ΔRD1 strain and *M. bovis* BCG led to 10–20% annexin V positivity and 10% or lower PI positivity (Figures S3A,B in Supplementary Material). Infection with ΔRD1 strain or *M. bovis* BCG led to retention of greater number of adherent cells at MOI 50 (Figures 4B,D) as a result of significantly lower necrosis (Figures S3C,D in Supplementary Material). We found fewer number of adherent cells remaining at MOI 1 in case of ΔRD1 compared to the wild-type strain (Figure 4B), probably due to higher apoptosis, as LDH release assays indicated no difference in necrosis at this MOI (Figure S3C in Supplementary Material). High MOI-dependent increase in TG synthesis did not occur during infection with ΔRD1 strain or BCG to the extent it did in H37Rv infection (Figures 4C,E). Increased TG synthesis was consistent with increase in incidence of larger LDs and higher total cellular BODIPY493/503 fluorescence in macrophages infected with H37Rv compared to H37RvΔRD1 at MOI 50 (Figures S3E–G in Supplementary Material). To rule out the possibility of these differences arising out of differential uptake of H37Rv and H37RvΔRD1, we quantified mean bacterial fluorescence intensity per cell in both conditions at all MOIs. While the mean bacterial fluorescence intensity per cell increased with increasing MOI, it was not different between macrophages infected with H37Rv and H37RvΔRD1 infections at the same MOI (Figure S3H in Supplementary Material). This further validates the role of RD1-mediated necrosis in increasing TG accumulation as a bystander response to necrosis.

To rule out the possibility that RD1 regulates fatty acid uptake by macrophages independent of necrosis, we used IM54, an inhibitor of H_2_O_2_ induced necrosis (28), to inhibit necrosis in Mtb infected THP1 macrophages at MOI 50. IM54 at 20 µM increased number of adherent cells by 2.5-folds when compared with the vehicle control (Figure 4F). This was consistent with a dose-dependent inhibition of necrosis by IM54, where 20 µM concentration reduced cell death from 70 to 40% at MOI 50^24h^ (Figure S3I in Supplementary Material). This concentration of IM54 led to 2.5-fold reduction in TG synthesis (Figure 4G), confirming a role for infection induced necrosis to be a player in stimulating TG synthesis in bystander macrophages.

### Development of an *Ex Vivo* Model of NAFMs

**Figure 5:**
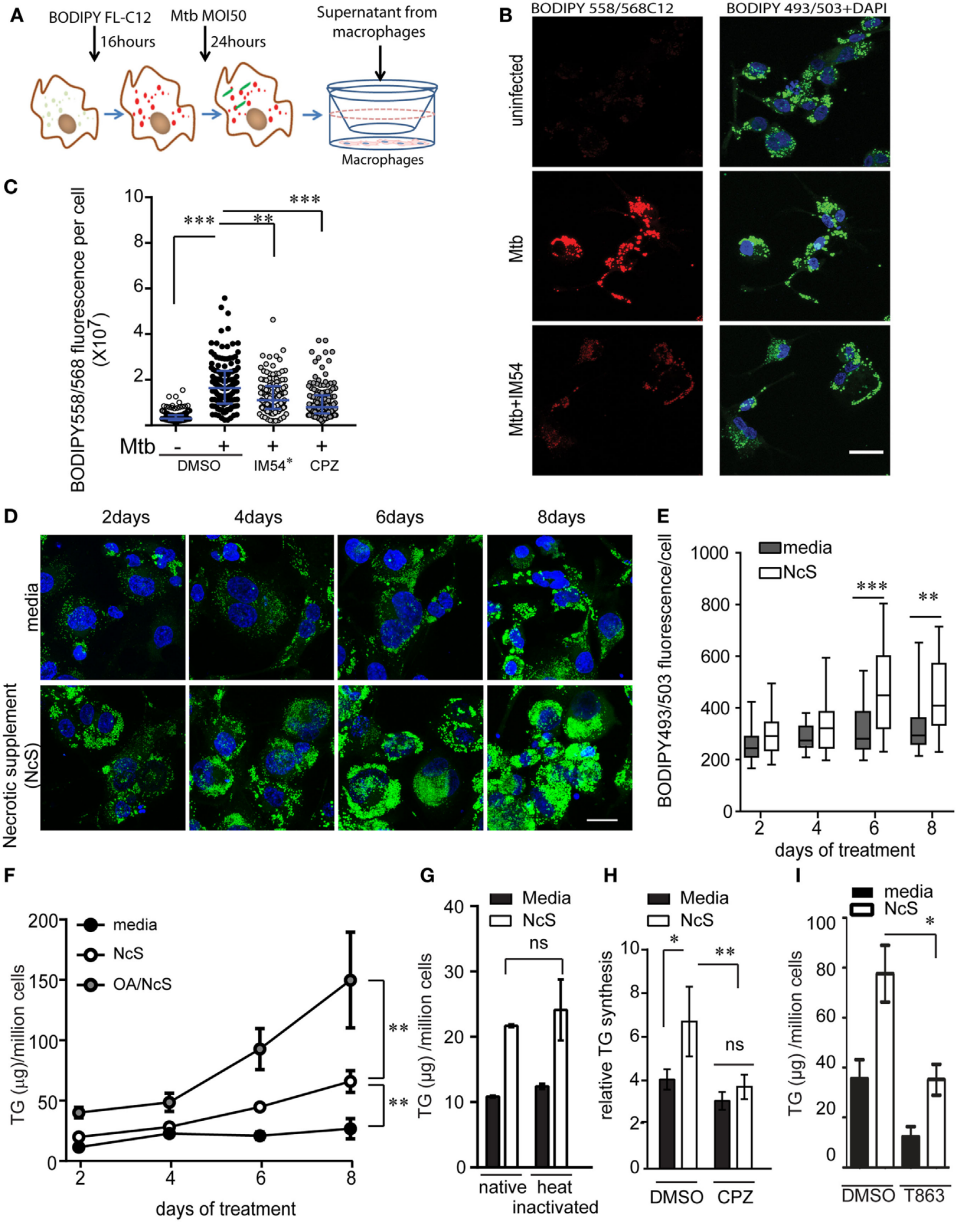
*Ex vivo* model of necrosis-associated foamy macrophages. **(A)** Schematic representing experimental setup for supernatant transfer from BODIPY558/568C12 labeled infected THP1 macrophages. **(B)** BODIPY 493/503 and DAPI staining in THP1 macrophages treated with supernatant from uninfected or *Mycobacterium tuberculosis*-infected THP1 macrophages labeled with BODIPY558/568C12 prior to infection. Scale bar = 25 μm. **(C)** Quantification of BODIPY558/568 fluorescence per recipient cell from experiment shown in **(A)**. IM54* indicates treatment of donor cells with IM54 during infection while chlorpromazine (CPZ) was added to recipient cells. DMSO was used as a vehicle control at 0.1% vol/vol. Data are from three independent experiments, with approximately 100 cells per group. **(D)** BODIPY493/503 (green) and DAPI (blue) stained THP1 macrophages at days 2, 4, 6, and 8 post treatment with media or necrotic cell supplement (NcS). Scale bar = 25 μm. **(E)** Quantification of total cellular BODIPY493/503 signal from a representative experiment. **(F)** TG estimation from THP1 macrophages post 2, 4, 6, and 8 days of differentiation. Gray circles represent NcS prepared from oleic acid pre-treated cells. 150 µM bovine serum albumin-conjugated oleic acid was added to cells 48 h prior to NcS preparation. Data are mean ± SEM from three independent experiments. **(G)** TG estimation from THP1 macrophages at 8 days post differentiation with normal media, NcS, or heat-inactivated normal media or heat inactivated NcS. Data are mean ± SD from triplicate wells. Data are representative of two independent experiments. **(H)** TG synthesis over 2 days in THP1 macrophages in response to heat inactivated NcS or media in the presence of DMSO or chlorpromazine (CPZ). Data are mean from three independent experiments, normalized to 5 h time point for normal media. **(I)** TG estimation in macrophages exposed to media or NcS after 8 days of treatment in the presence of DMSO (vehicle control) or T863 (10 µM). Data are mean ± SEM from three independent experiments. Kruskal–Wallis test derived *p*-values are shown in **(C,E)**. Linear regression analysis for slopes was done in **(F)**. *T*-test derived *p*-values are indicated in **(E,G–I)** (***p < 0.001, **p < 0.01, *p < 0.05, ns, not significant).

To further understand if lipids released from necrotic cells were contributing to the TG pool of uninfected or bystander macrophages, we employed a transwell assay in which uninfected THP1 macrophages were seeded in the lower chamber and supernatant from MOI 50^24h^ infected cells was added to the upper chamber (Figure 5A). The transwell prevented effects due to bacterial infection of recipient (bystander) cells (Figure S4A in Supplementary Material). To chase the lipids from the donor cells, they were labeled with BODIPY558/568-C12 FA prior to infection. BODIPY558/568-C12 FA labeled lipids from MOI 50^24h^ infected cells could be assimilated into the neutral lipid pool of uninfected cells across the transwell (Figures 5A,B). The transwell allowed transfer of lipids with a lower tendency for most neutral lipids such as TGs to pass through while more polar lipids such as diacylglycerides, phospholipids, and fatty acids passed through more readily (Figure S4B in Supplementary Material). While free fatty acids could traverse the filter, they were the least abundant species and did not increase during the course of the transwell experiment, suggesting lipid esterified BODIPY558/568-C12 to be the major source of the label in this experiment. Prevention of necrosis with IM54 prevented transfer of labeled lipids to the LDs of recipient cells, confirming that lipids of cells undergoing necrosis can provide a source of fatty acids for TG synthesis in bystander cells (Figures 5B,C). For recipient macrophages to acquire fatty acids from lipids for new synthesis, these lipids must first undergo lysosomal degradation (29). CPZ, an inhibitor of lysosomal lipases (30), could significantly inhibit label transfer, confirming a role for new TG synthesis from incoming lipids that included diacylglyceride and TG (Figure 5C).

The above experiments suggested that lipids released from necrotic cells may be sufficient to develop “necrosis-associated foamy macrophages” *ex vivo*. To further test if exposure to necrotic cells was sufficient to stimulate TG accumulation in bystander macrophages, we stimulated healthy THP1 macrophages with mechanically necrotized macrophages (NcS). NcS generated by mechanical disruption of healthy macrophages contained 1.4-fold higher TG than media, while protein and glucose concentration of both media and NcS was similar (Figure S4C in Supplementary Material). We evaluated the ability of NcS to enable TG storage in recipient macrophages at a donor cell to recipient ratio of 2:1. This ratio simulated the scenario of Mtb infection at MOI 50, wherein at least two-thirds of the cells were lost for a lipogenic response to be observed in the remaining one-third bystander macrophages. NcS was able to stimulate accumulation of LDs in healthy macrophages in a time-dependent manner (Figures 5D,E), corresponding to a temporal increase in total cellular TG (Figure 5F). NcS-stimulated cells increased their TG levels in a time-dependent manner with a 3- to 5-fold increase by day 8 compared with day 0 while normal media treated cells exhibited only 1.2-fold increase in TG content during the same time (Figure 5F). Dilutions of the NcS led to decreased LD abundance in recipient cells (Figure S4D in Supplementary Material) while increasing the lipid load of donor cells by prior treatment with oleic acid (31) further increased the TG content of recipient cells by twofolds in a time-dependent manner (Figure 5F), validating a role for exogenous lipid in increasing TG content of recipient macrophages. Heat inactivation of the NcS failed to abrogate TG accumulation in recipient cells, suggesting active process from the donor not likely to contribute to the phenomenon (Figure 5G). Fatty acids could be mediating this response if they are first degraded by the cellular machinery as majority of cellular fatty acids are present in esterified form. Inhibition of lysosomal lipases by CPZ led to impairment in NcS stimulated TG synthesis (Figure 5H), consistent with the mode of lipid uptake during high MOI Mtb infection. While exogenous lipids were assimilated in the presence of NcS, exogenous glucose uptake was in fact decreased in response to NcS (Figure S4E in Supplementary Material). Because glucose feeds into *de novo* fatty acid synthesis, we speculated that *de novo* FA synthesis might not be required for NAFMs formation. Inhibition of FA synthase with C75 did not lead to any change in total TG levels upon stimulation with NcS (Figure S4F in Supplementary Material) even though it led to 80% inhibition of *de novo* FA synthesis (Figure S4G). NcS-dependent TG synthesis was abrogated by T863, an inhibitor of DGAT1 (32), verifying the role of endogenous esterification of incoming lipid-derived FA rather than *en masse* storage of the excess exogenous TG (Figure 5I). TG-rich macrophages generated by exposure to NcS are hereafter referred to as NAFMs.

### TG Storage in Human Macrophages Upregulates Infection Induced Pro-Inflammatory Response

**Figure 6:**
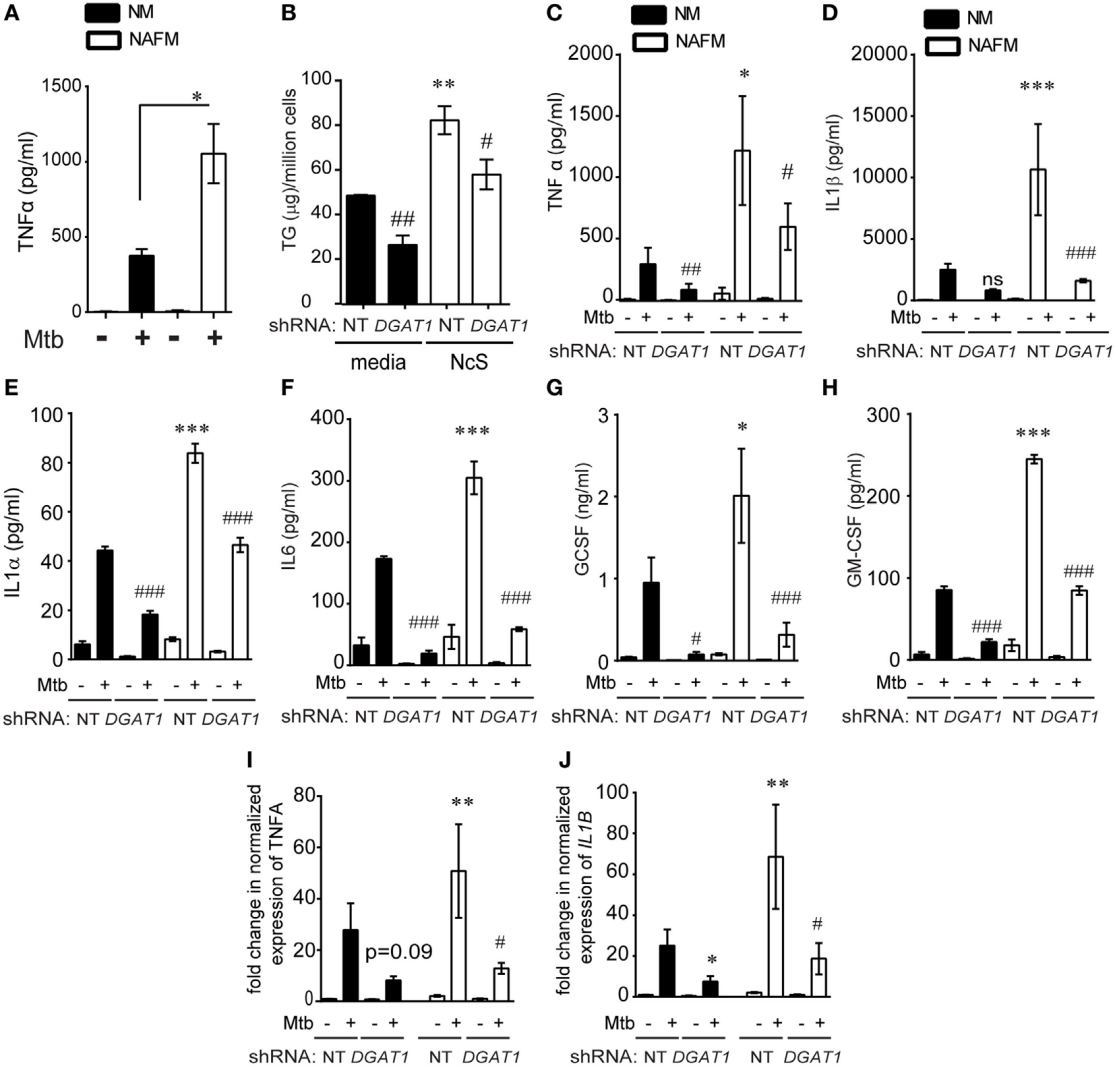
DGAT1-mediated TG synthesis is responsible for the heightened inflammatory response to *Mycobacterium tuberculosis* (Mtb) infection in foamy macrophages. **(A)** TNFα release measured by ELISA in supernatants from THP1 cells that were normal media treated (termed as normal macrophages or NM) or treated with necrotic cell supplement (NcS) for 8 days [termed as necrosis-associated foamy macrophages (NAFMs)] at 24 h after infection with Mtb at multiplicity of infection (MOI) 50 for 3 h. Data are mean ± SEM from three independent experiments (*p < 0.05). **(B)** TG estimation from NT and *DGAT1* knockdown THP1 macrophages at 8 days post differentiation with media or NcS. Data are mean ± SEM from triplicate experiments. **(C)** TNFα release measured by ELISA in supernatants from NM and NAFM of NT and *DGAT1* knockdown THP1 macrophages at 24 h post infection with Mtb at MOI 50 for 3 h. Data are mean ± SEM from three independent experiments. **(D–H)** Luminex-based detection of indicated cytokines in supernatants from Mtb-infected NM and NAFM of *DGAT1* and non-targeting shRNA expressing macrophages. Data are mean ± SEM from five to six wells from two independent experiments. Transcript abundance of *TNFα* **(I)** and *IL1B* **(J)** normalized to that of PPIA in NMs and NAFMs of NT and DGAT1 shRNA expressing THP1 macrophages. Data are mean ± SEM from three experiments (*** or ^###^*p* ≤ 0.01, ** or ^##^*p* ≤ 0.01, * or ^#^*p* ≤ 0.05, ns, not significant). * represents comparison between NAFM and NM and # represents comparison between *DGAT1* knockdown and non-targeting control (NT) expressing macrophages for each group. See also Figure S5 in Supplementary Material.

During infection, necrosis is a progressive phenomenon arising due to increased bacterial burden in infected macrophages, wherein NAFMs would form as a bystander response and then subsequently get infected. Our *in vivo* data suggested possibility of increased inflammatory response in oil red O positive foamy macrophages. To investigate the role of TG content in altering the inflammatory response, we questioned whether infection of *ex vivo* generated NAFMs, exhibit differences in immune response to Mtb infection. We found that Mtb infected NAFMs (THP1 derived) released 1.5- to 3-fold higher TNFα compared with infected THP1 NMs (Figure 6A) while neither did NAFMs exhibit a heightened basal secretion of TNFα (Figure 6A) nor did the NcS media itself contain detectable levels of TNFα (data not shown). We questioned if this increase was due to increased bacterial uptake in foamy macrophages. We found no difference between NM and NAFM in the uptake of Mtb, suggesting inherent potential of NAFMs to mount a higher pro-inflammatory response independent of bacterial burden (Figure S5A in Supplementary Material). These infection conditions led to approximately 40% necrotic cell death (Figure S5B in Supplementary Material), which we previously found to be insufficient for necrosis-triggered TG synthesis (Figures 4C–E; Figure S3C in Supplementary Material), suggesting priming of cells during NAFM differentiation for the heightened responses rather than necrosis induced TG synthesis during infection. Primary human MDMs also exhibited increased staining with BODIPY493/503 and 1.2- to 2-fold increase in cellular TG in response to NcS over a period of 8 days (Figures S5C,D in Supplementary Material). Three out of four donors also exhibited increase in infection induced TNFα release upon differentiation to NAFMs (Figure S5E in Supplementary Material).

To further test if cytokine production in response to infection was proportional to intracellular TG levels, we differentiated DGAT1 shRNA and control shRNA expressing THP1 cells with NcS and then sought to measure release and expression of TNFα in response to Mtb infection. *DGAT1* knockdown cells exhibited approximately 50% decrease in TG levels in case of NMs and 30–40% decrease in case of NAFMs (Figure 6B).

*DGAT1* knockdown led to approximately 50% decrease in infection induced TNFα in the supernatant in case of NM (Figure 6C). To check a broader range of cytokines, chemokines, and growth factors that are expressed by macrophages in response to Mtb infection, we performed a luminex multiplex assay. We observed that besides TNFα, IL-1β, IL-1α, IL-6, GCSF, and GMCSF release upon Mtb infection was 2- to 2.5-fold higher from NAFMs compared to NMs (Figures 6D–H). Infection induced release of all of these cytokines decreased upon depletion of *DGAT1* transcripts (Figures 6D–H) in macrophages despite their prior stimulation with NcS, confirming the role of TGs in the inflammatory response to infection. To further verify whether *DGAT1* regulated levels of these key cytokines and growth factors at the level of secretion or transcription, we quantified transcript abundance for *TNFα* and *IL1β*. Infection-induced *TNFα* transcript abundance was reduced by approximately 60–80% upon *DGAT1* knockdown (Figure 6I). *IL1β* transcript abundance was also found to be 40–80% lower in NMs and 60–80% lower in NAFMs upon depletion of *DGAT1* expression (Figure 6J). Cholesterol is also known to regulate inflammation (33) but estimations from NMs and NAFMs showed that neither NcS stimulation nor DGAT1 silencing altered total cellular cholesterol levels (Figure S5F in Supplementary Material), delinking the possibility of cholesterol mediated control of inflammation in this model. Therefore, the pro-inflammatory response of human macrophages to Mtb was indeed dependent on their intracellular TG abundance. Surprisingly, we found no significant differences in the uptake or growth of Mtb in NM and NAFM, whether in the wild-type or *DGAT1* knockdown background (Figures S5G–I in Supplementary Material). Therefore, intracellular TG levels regulate specific innate immune responses during Mtb infection without affecting growth of the intracellular bacilli.

## Discussion

**Figure 7:**
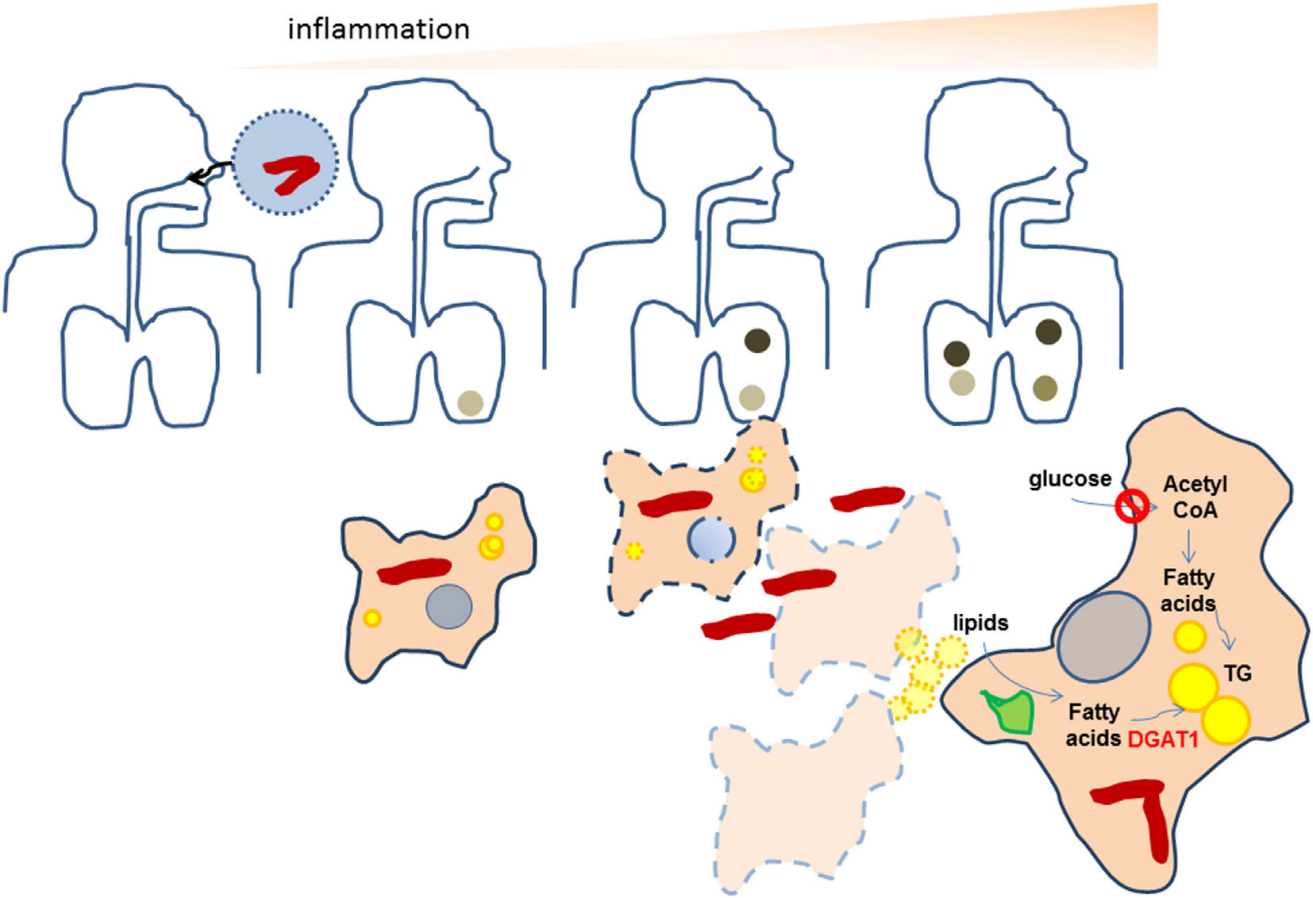
Proposed scheme for the amplification of inflammation as a result of neosynthesis of triglycerides (TGs) in bystander foamy macrophages in TB. Bacteria are indicated as red rods while lipid droplets are indicates as yellow spheres. Darkened regions in the lung indicate granulomas. During initial infection of macrophages with *Mycobacterium tuberculosis* (Mtb), low levels of TGs are present in infected macrophages. The inflammatory response of macrophages recruits immune cells that help in granuloma formation. As bacteria multiply, both host and bacterial factors may synergize to bring about necrosis of infected cells, leading to formation of necrotic granulomas. Macrophages surrounding the necrotic center differentiate to TG-rich (yellow) foamy macrophages using lipids available from the necrotic cells, by first degrading them using lysosomal lipases (green). In doing so, they downregulate their glucose uptake and do not rely on *de novo* fatty acid synthesis for the increase in TG levels. Upon infection with Mtb, these necrosis-associated foamy macrophages release higher levels of pro-inflammatory mediators. The pro-inflammatory response of macrophages is dependent on the increased TG levels. The evolution of granulomas to necrotic granulomas, thereby amplifies the inflammatory response.

This study, for the very first time, illustrates the role of macrophage TG levels in the pro-inflammatory response of foamy macrophages during Mtb infection. Here, we demonstrate that Mtb-induced necrosis stimulates TG accumulation in foamy macrophages is a bystander response. Once TG loaded cells encounter bacilli, they mount a higher pro-inflammatory response than cells with lower TG levels. We demonstrate that lysosomal degradation of incoming lipids is important for neosynthesis of TGs in a DGAT1-dependent manner in the bystander cells. In this process, the bystander foamy cells reduce their uptake of glucose and do not rely on *de novo* fatty acid synthesis for synthesis of the new TG. This model is able to explain the *in vivo* tripartite association of increased inflammatory response, presence of TG-rich foamy macrophages, and necrotic granulomas and is able to provide insights into the relevant carbon sources that polarize macrophages into a metabolically altered state in TB (Figure 7). We demonstrate that RD1 dependent necrosis in the context of TB infection is crucial for this, but can be generalized to necrosis independent of infection also. This suggests that TG accumulation in macrophages in the context of other necrotic pathologies might also be important for sustaining inflammation to infectious insult. These findings could be relevant in the case of necrotic atherosclerotic plaques and necrotic infections.

Triglycerides have been considered inert storage lipids; our data reveals that TG levels are important in titrating the inflammatory response to infection with Mtb. There are several possible mechanisms whereby this could happen. First, metabolites of TGs, namely fatty acids and glycerides, could regulate inflammation (34–37). Eicosanoids derived from fatty acid precursors such as prostaglandin E2, lipoxins LXA4, and LTB4 are known mediators of the inflammatory response in TB infection (38, 39). PGE2 in particular is important for IL1β expression in monocytes and macrophages (2, 40). Antagonistic to PGE2, which prevents necrosis (41), H37Rv induced LXA4 production promotes necrosis (38). By contrast, the avirulent strain H37Ra induces PGE2 production which inhibits LXA4 production, thereby preventing necrosis (38). PGE2 is also a negative regulator of type I IFN induction (2) and early Type I IFN response during murine infection promotes cell death (42). Our investigations thus far have revealed that infection induced production of prostaglandin E2 or of infection-induced necrosis are not, however, regulated by TG levels (data not shown) even though IL1β expression in our model is dependent on TGs. This suggests that lipids other than TGs may be more important sources of PGE2 production during infection. A further investigation into all possible eicosanoid classes generated by macrophages upon Mtb infection is warranted to understand the role of TGs in inflammation. Besides eicosanoids, sphingolipids and phospholipids also share common metabolites of TG degradation. These lipid classes are also known activators of pro-inflammatory signal transduction within cells (43, 44). While these pathways involve the classical outside-to-inside signaling, signal transduction within the cell could also be regulated by specific proteins that interact with LDs. ER and mitochondrial membranes act as signaling hubs that modulate NOD2 and inflammasome, respectively (45, 46). Recently, viperin was demonstrated to mediate recruitment of TLR adaptors to LDs in plasmacytoid dendritic cells (47). It remains to be understood whether and how LD surface regulates signal transduction in infected macrophages.

Previous studies have suggested a role for mycobacterial lipids in stimulating macrophage neutral lipid accumulation (48, 49); while the carbon source for these lipids itself is not mycobacterial lipids, we suspect that in addition to necrosis, these lipids could additionally stimulate lipogenic signaling in infected cells. Human macrophages cultured *in vitro* tend to store TGs; therefore, evaluating modulation of the TG content upon infection requires the use of pulse chase-based assays while steady-state level metabolite analysis without flux analysis may prove to be misleading. Thus, this study provides evidence for unaltered rate of TG synthesis under conditions where cell death is minimal. Furthermore, our data suggest that cells that are bystanders of necrosis increase their TG content further if the donor TG content is increased. This is consistent with previous work describing the ability of TG-rich very low-density lipoproteins to increase macrophage TG content (50). Mouse models of Mtb infection have been extensively used to identify mechanisms of foamy macrophage formation in Mtb infection. While some studies report inhibition of autophagy-mediated lipid degradation regulated by microRNA miR-33 (51), others have reported a TLR2-dependent increase in lipid synthesis genes upon Mtb infection to be responsible for this process (52). A recent study suggested that IFNγ and Mtb synergize for foamy macrophage formation using mouse bone marrow-derived macrophages (53). However, the human TB and guinea pig models of TB suggest a possible role for incident pathology in driving foamy macrophage formation. While Mtb induced necrosis is operative in C57BL6 model of infection (54), it is not evident as “necrotic lesions” unless probed with specific markers. Therefore, ascertaining inherent variability in granuloma types and the role of necrosis has probably not been addressed in the resistant mouse models used in the above described studies. Moreover, building a causal role for necrosis-mediated foamy macrophage formation *in vivo* is challenging as strains such as RD1 deletion mutant induce largely lymphocytic granulomas (55) and inhibitors of Mtb induced cell death during *in vitro* experiments have shown limited potency *in vivo* (56). Based on our data, and studies summarized above, we suggest that any primary trigger that leads to increase in cellular TG may be further amplified to increase the abundance of TG-rich foamy macrophages once necrosis takes place. Our study here recapitulates the strong correlation between necrosis and foamy macrophage formation found in human TB (48) and helps explain the coincidence of spatially polarized expression of TNFα (18). Importantly, our work provides a new *ex vivo* model of foamy macrophages which can be used in future for studies pertaining to host response and antibiotic sensitivity.

*Mycobacterium tuberculosis* evades host immunity and adapts to the host’s lipid rich environment (57, 58). Therefore, the importance of lipids for mycobacterial growth in macrophages has been a major area of investigation (59). Cholesterol utilization pathways of Mtb and host fatty acids are essential for bacterial survival in macrophages (60, 61). An important paradigm shift that appears from our work, consistent with that recently reported by the Stanley group using mouse macrophages (53), is that Mtb growth is not dependent on host TG accumulation by DGAT1. Phospholipids, sphingolipids, and free fatty acids could be the other carbon sources utilized by Mtb within macrophages. Alternatively, TG synthesis by DGAT2 could also contribute to enough TGs such that bacterial growth can be sustained. It remains to be ascertained whether TG accumulation *in vivo* alters Mtb growth. These findings can help us understand the contribution of foamy macrophages to disease and could possibly be coupled with antimicrobial drugs as a host-directed therapy to decrease host damage and synergistically help clear infection.

Lipid metabolism in macrophages plays a critical role in inflammation during metabolic disorders (62), atherosclerosis (63), and neurodegeneration (64). Increased TG accumulation in metabolic disorders has been reported to be majorly diet induced, where excess dietary fatty acids are stored into TG in the form of cytosolic LDs (20). Increased inflammation in a murine model of diet-induced obesity can be reversed by overexpression of DGAT1 in macrophages (65). Similarly, increasing fatty acid degradation by overexpressing CPT1, the rate-limiting enzyme in fatty acid oxidation, also plays a protective role against lipid-induced inflammation (66). While it is tempting to speculate that there may be species-specific regulation of lipid metabolism, as suggested earlier (67, 68), presentation of cellular lipids in the context of necrosis versus circulating free fatty acids and lipids as under condition of dietary excess might underlie the distinction between our model and these studies. An in-depth understanding of the mechanism involved in uptake of lipids from necrotic cells would be important in regulating functions of the foamy macrophage. Processes such as efferocytosis, phagocytosis, or receptor-mediated endocytosis could be involved. Our preliminary investigations ruled out efferocytosis or phagocytosis to be involved (data not shown). Finally, culmination of uptake into TG synthesis is important for attainment of a state primed for pro-inflammatory responses. Future work is required to assess the role of lipid uptake and synthesis machinery in regulating inflammation in animal models of TB.

## Ethics Statement

This study was carried out in accordance with the recommendations of the Institutional Human Ethics Committee. The protocol was approved by the Institutional Human Ethics Committee. All subjects gave written informed consent in accordance with the Declaration of Helsinki. This study was carried out in accordance with the recommendations of Care and Use of Laboratory Animals issued by the Committee for the Purpose of Supervision of Experiments on Animals (CPCSEA) under the Prevention of Cruelty to Animals Act 1960 including amendments introduced in 1982 by Ministry of Environment and Forest, Government of India. The protocol was approved by the Institutional animal Ethics Committee.

## Author Contributions

Conceptualization, supervision, funding acquisition: SG. Methodology: SG and NJ. Investigation: NJ and SD. Microscopy: NJ, SD, KS, AN, DM, and SG. Validation: DM, KS, AN, and PB. Analysis: NJ, DM, and SG. Resources: GK, AT, and SG. Writing: NJ and SG.

## Conflict of Interest Statement

The authors declare that the research was conducted in the absence of any commercial or financial relationships that could be construed as a potential conflict of interest.

## Abbreviations

Mtb, *Mycobacterium tuberculosis*; TB, tuberculosis; MOI, multiplicity of infection; NcS, necrotic cell supplement; NAFMs, necrosis-associated foamy macrophages.
