# Supplementary figures and images for: Necrosis Driven Triglyceride Synthesis Primes Macrophages for Inflammation During Mycobacterium tuberculosis Infection
Source: Front Immunol. 2018 Jul 3;9:1490. doi: 10.3389/fimmu.2018.01490 (PMC6037689; doi:10.3389/fimmu.2018.01490)

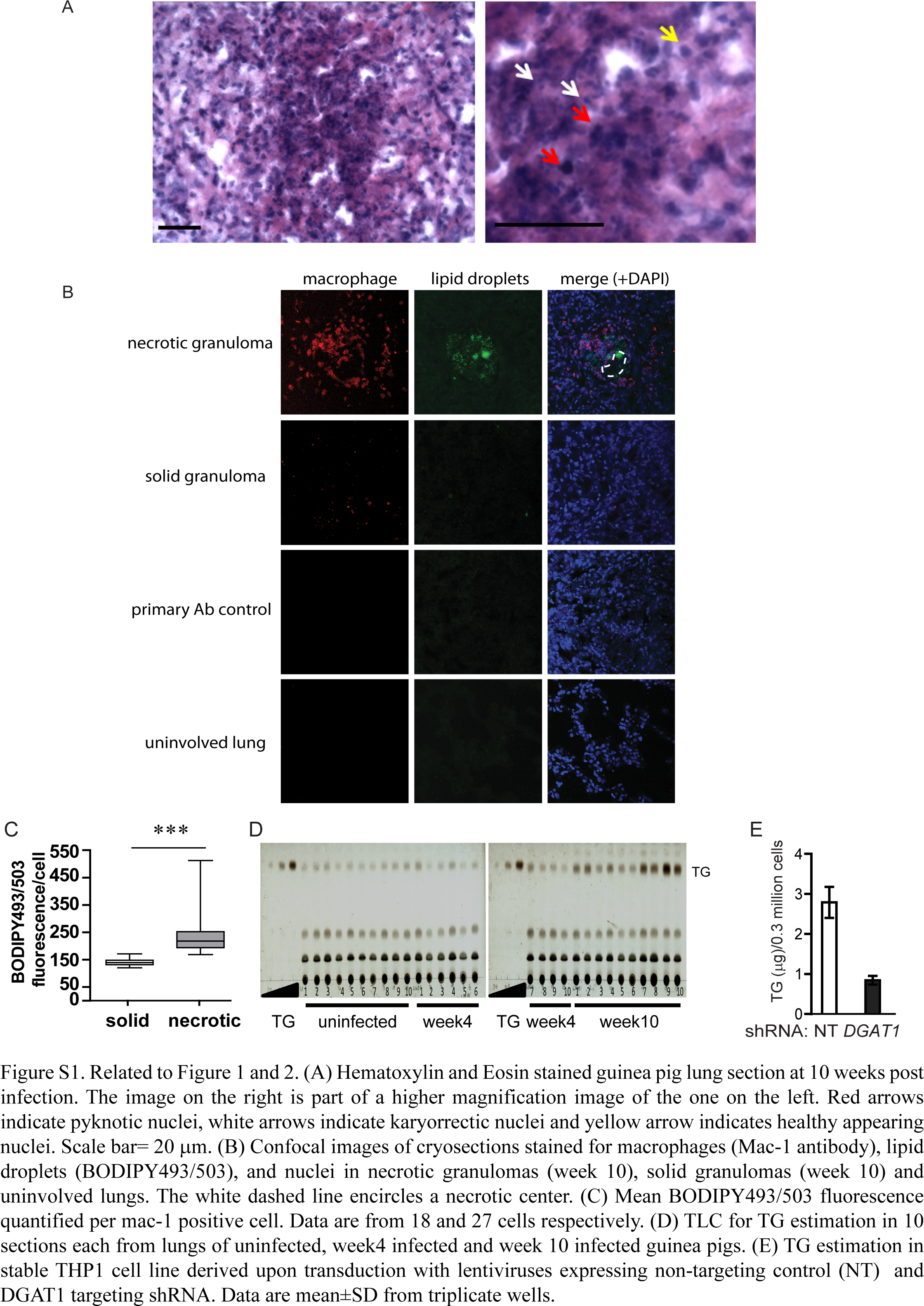

Supplement: Supplementary file 1 [file Image_1.tif]

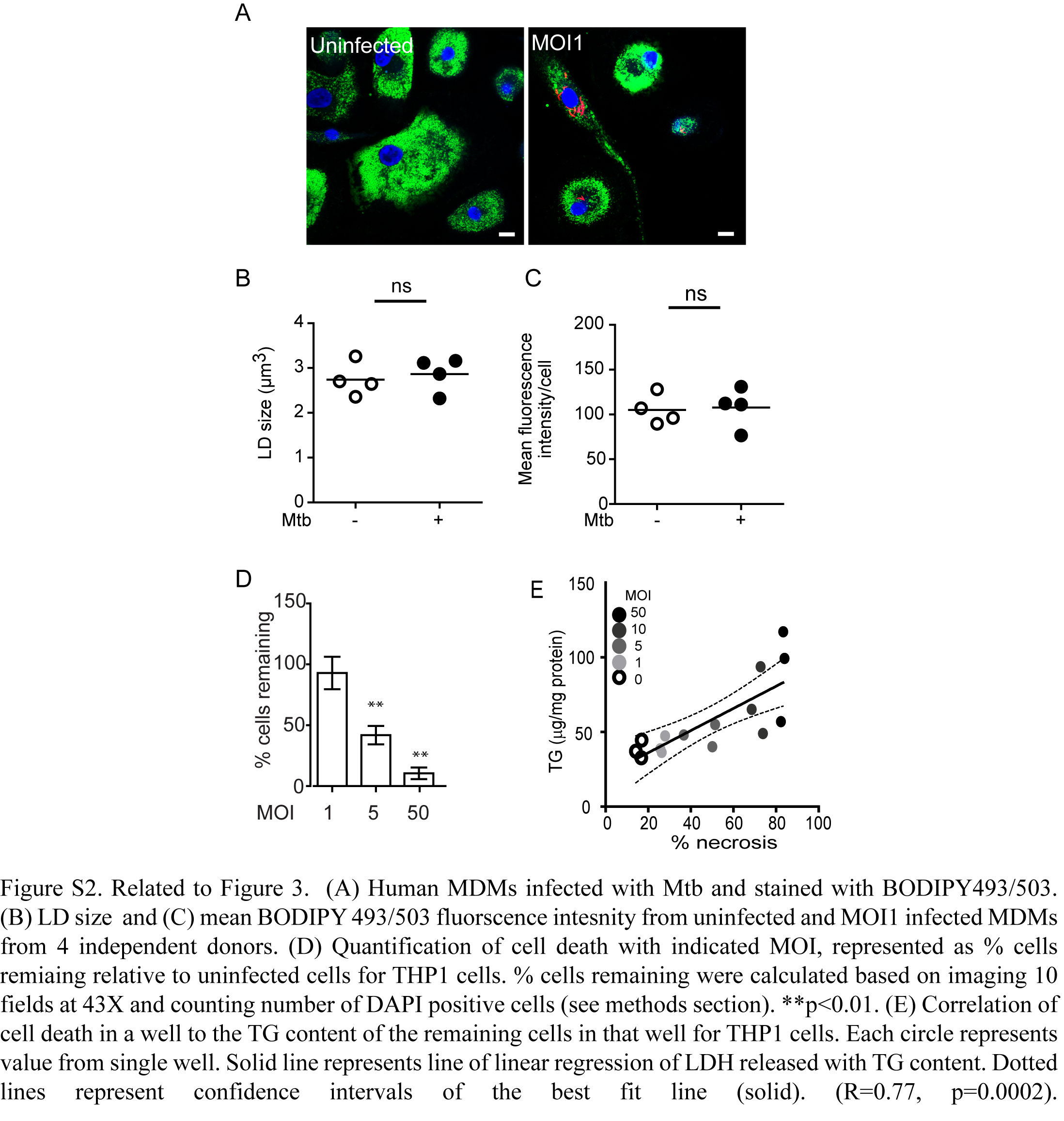

Supplement: Supplementary file 2 [file Image_2.tif]

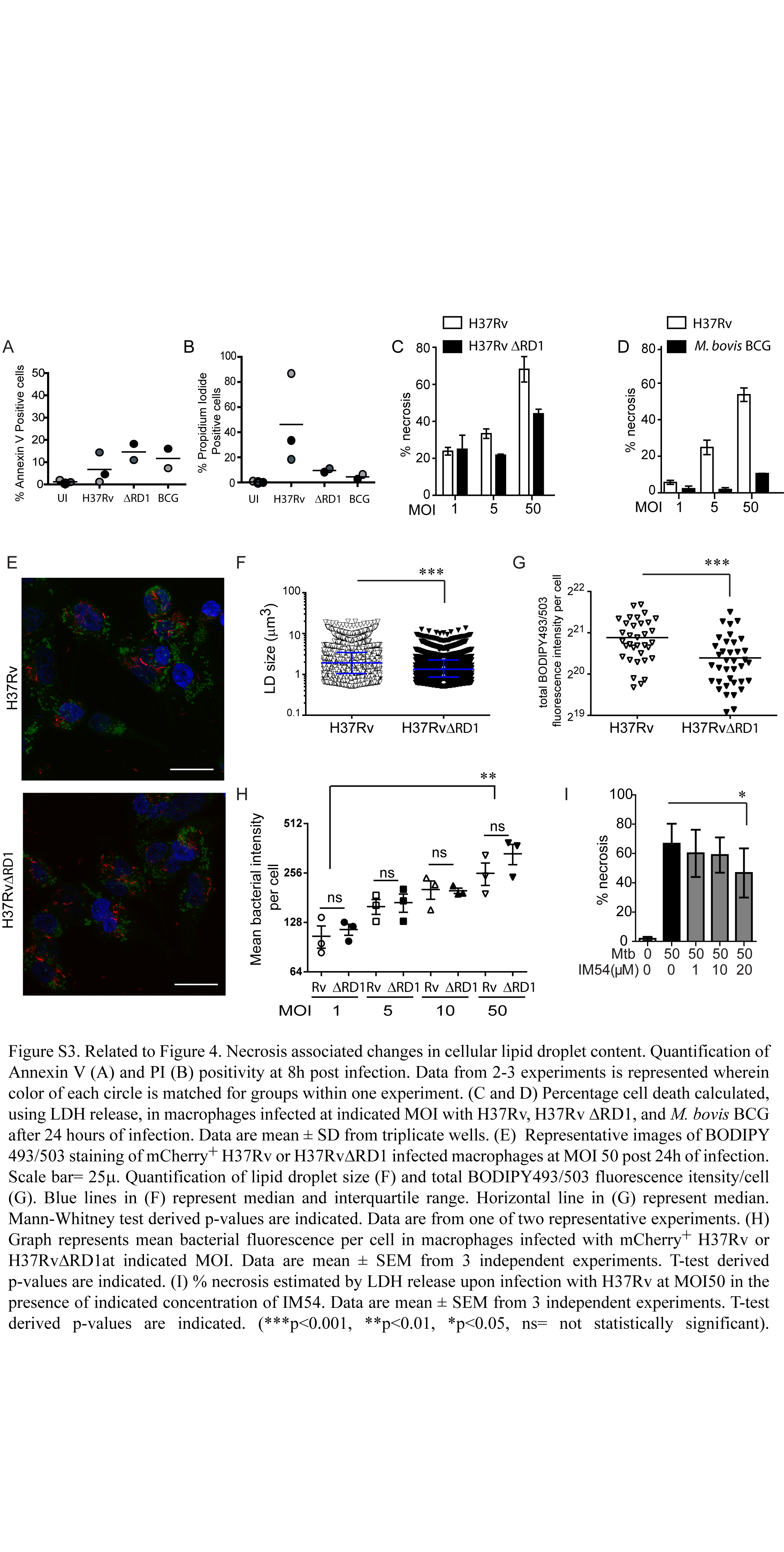

Supplement: Supplementary file 3 [file Image_3.tif]

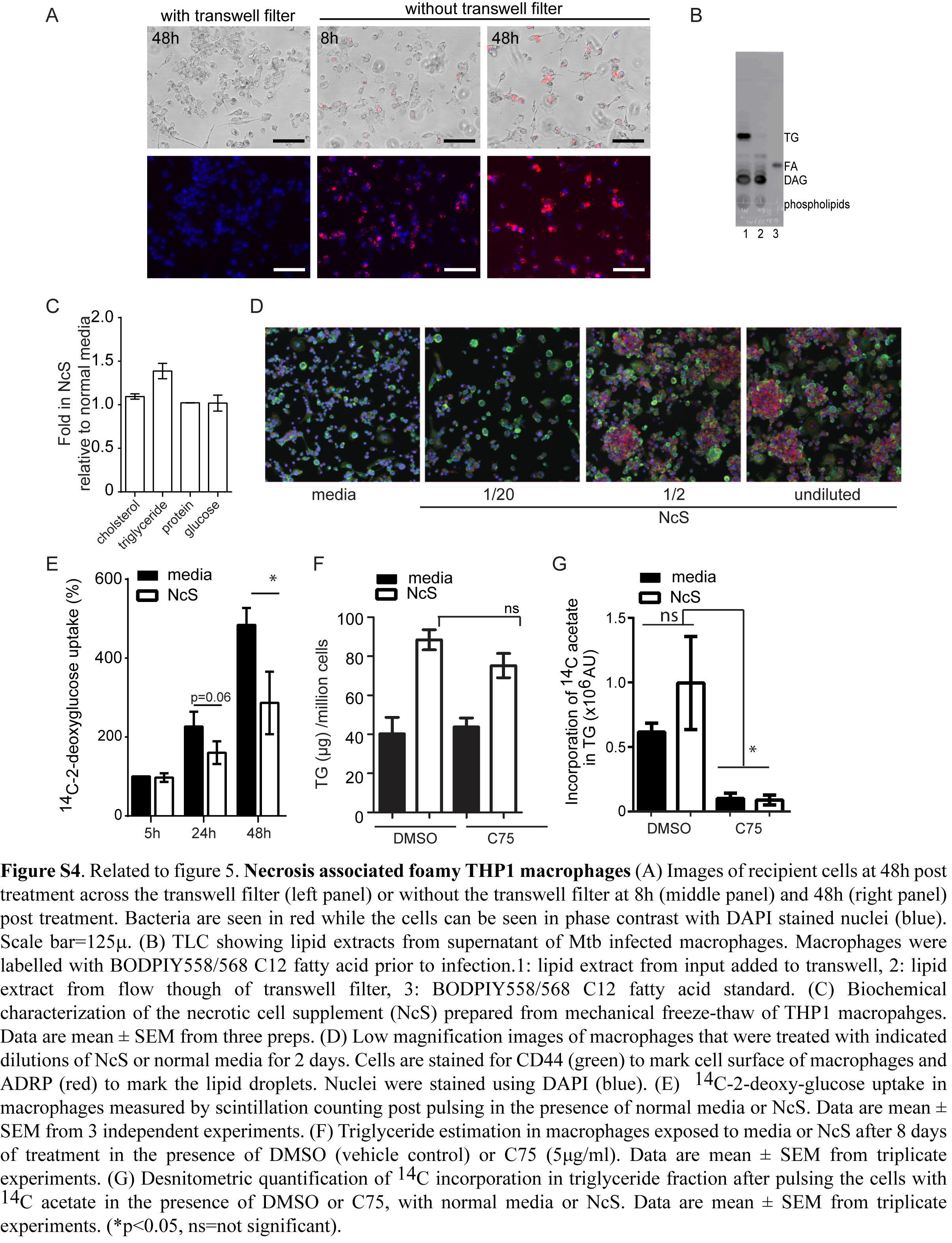

Supplement: Supplementary file 4 [file Image_4.tif]

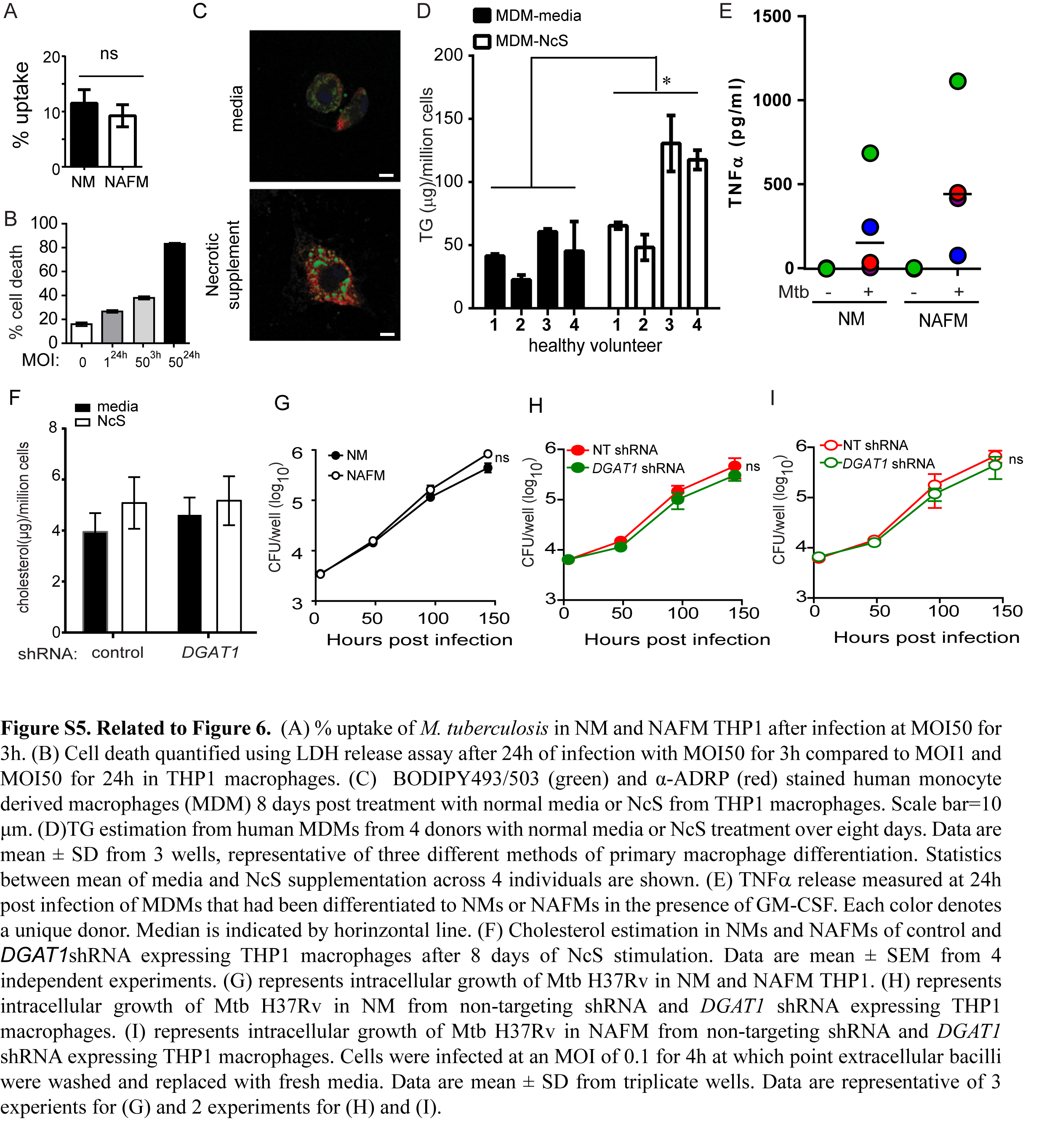

Supplement: Supplementary file 5 [file Image_5.tif]
